# Exposure to Environmental Tobacco Smoke: a Modifiable Factor of Hospitalization and Severity of Acute Bronchiolitis

**DOI:** 10.1016/j.jped.2026.101629

**Published:** 2026-10-09

**Authors:** Lorena Bermúdez-Barrezueta, Wysali Trapiello Fernández, Irune Torres Ballester, Pilar Uribe-Reina, Pablo López-Casillas, Sergio Madero-Juez, Hermenegildo González García, María Asunción Pino-Vázquez

**Affiliations:** a University of Valladolid, Faculty of Medicine, Department of Pediatrics, Valladolid, Spain; b Hospital Clínico Universitario de Valladolid, Department of Pediatrics, Division of Pediatric and Neonatal Intensive Care, Valladolid, Spain; c Hospital Clínico Universitario, Laboratory Medicine Department, Valladolid, Spain; d Hospital Clínico Universitario de Valladolid, Department of Pediatrics, Valladolid, Spain; e Hospital Universitario Río Hortega, Department of Cardiology, Valladolid, Spain; f Hospital Clínico Universitario de Valladolid, Department of Pediatrics, Division of Pediatric Hematology and Oncology, Valladolid, Spain

**Keywords:** Acute bronchiolitis, Cotinine, Environmental tobacco smoke

## Abstract

**Objective:**

The objective was to evaluate the associations of environmental tobacco smoke exposure with acute bronchiolitis and its severity among hospitalized children.

**Methods:**

This case-control study with prospective follow-up was conducted at a tertiary care hospital (February 2017-December 2021). Infants hospitalized for bronchiolitis were enrolled as cases, and hospitalized children < 2 years of age without respiratory disease were recruited as controls. Serum cotinine was measured in all participants. Demographic and clinical data and household smoking habits were collected through parental interviews. Logistic regression was used to assess the association between environmental tobacco smoke exposure and bronchiolitis. Additionally, whether serum cotinine concentration was associated with admission to the pediatric intensive care unit and length of hospital stay in infants with bronchiolitis was investigated.

**Results:**

In total, 251 infants (151 cases, 100 controls) were included; 52.6% of participants were exposed to environmental tobacco smoke, with a higher prevalence among the bronchiolitis group (59.6%vs. 42%; p = 0.006). Multivariate analysis revealed that environmental tobacco smoke exposure (OR: 1.93; 95% CI: 1.04–3.56) and prenatal maternal smoking (OR: 3.49; 95% CI: 1.22–9.95) were independently associated with hospitalization for bronchiolitis. Among the cases, serum cotinine levels > 5 ng/mL predicted prolonged length of hospital stay (OR: 4.99; 95% CI: 1.70–15.7), whereas prenatal smoking increased the risk for pediatric intensive care unit admission (OR: 4.73; 95% CI: 1.68–13.3).

**Conclusions:**

Pre- and postnatal environmental tobacco smoke exposure was associated with bronchiolitis and disease severity. Developing effective public health strategies to reduce such exposures among children is a priority.

## Introduction

Acute bronchiolitis is the most common lower respiratory tract infection among infants and is the leading cause of hospitalization in high-income countries. Although respiratory syncytial virus (RSV) is the primary etiological agent, acute bronchiolitis can also be caused by other viruses [1,2].

In Spain, approximately 100,000 episodes of acute bronchiolitis occur annually, with hospitalization rates of 20–25 per 1000 child-years among those under 2 years and 38 per 1000 child-years among infants under 12 months [3]. Despite progress in prevention and supportive care, the burden of bronchiolitis across all healthcare tiers remains substantial, representing a major public health concern worldwide [4,5].

Several factors are associated with an increased risk for bronchiolitis. Recent evidence suggests that exposure to environmental tobacco smoke (ETS) increases the incidence and severity of acute bronchiolitis [[6], [7], [8], [9]].

Although ETS exposure is a well-established risk factor for childhood respiratory diseases, [[10], [11], [12]] few studies have quantified this association using objective measurement methods [6,13]. Due to the methodological challenges of assessing ETS exposure, biomarker-based approaches may enhance diagnostic precision. Cotinine, a nicotine metabolite detected in the blood, urine, and saliva, has a half-life of 16–20 h, making it a more reliable biomarker than nicotine for assessing both active and passive smoking [14].

The primary objective of the present study was to evaluate the association between ETS exposure and acute bronchiolitis among hospitalized children, using serum cotinine as a biomarker of passive smoking. Additionally, the clinical course of children hospitalized for bronchiolitis was analyzed according to serum cotinine concentration and other factors.

## Material and methods

### Study design and population

This mixed observational case-control study with prospective follow-up of participants was conducted at the *Hospital Clínico Universitario de Valladolid* (Valladolid, Spain) between February 2017 and December 2021. Cases comprised consecutively enrolled infants hospitalized with acute bronchiolitis, defined as the first episode of respiratory distress with wheezing and/or crackles preceded by an upper respiratory tract catarrhal condition affecting children < 2 years of age [15]. The control group included children < 24 months of age who were admitted to the hospital during the same period for non-respiratory conditions and were selected using incidence-density sampling (3:2 case–control ratio). Serum cotinine levels were analyzed for all children. Patients transferred from other centers, who were hospitalized > 24 h, were excluded due to challenges in assessing recent ETS exposure. In the second phase of the study, infants admitted with acute bronchiolitis were prospectively monitored throughout hospitalization to determine the association between ETS exposure and the clinical course of the disease.

The study was approved by the clinical research ethics committee of the center, in accordance with the principles established in the Declaration of Helsinki. Written informed consent was obtained from the parents or legal guardians of all participants before inclusion.

### Sample size

The sample size was determined a priori from published estimates [9,16,17]. Assuming an alpha level of 0.05, 80% power, a 35% prevalence of ETS exposure among controls, an odds ratio (OR) of 2.2 for the association between ETS exposure and hospitalization for acute bronchiolitis, [17] and a case-control ratio of 1.5:1, the required sample size was determined to be 220 participants (132 cases, 88 controls). To account for the anticipated 10% attrition or non-evaluable data, target enrollment was increased to 245 participants (147 cases, 98 controls).

### Measurement of serum cotinine levels

Serum cotinine levels were measured within the first 24 h of hospitalization. After obtaining parental or guardian consent, a blood sample (0.5 mL) was obtained by cannulation of a peripheral venous line or by blood collection performed as part of routine clinical management, thus avoiding extra venipuncture. Samples were centrifuged and stored at −80 °C until analysis. Cotinine levels were quantified using liquid chromatography-mass spectrometry (LC-MS/MS; Reference International Laboratory, Spain). The laboratory-established limit of detection was 2 ng/mL, and concentrations at or above this threshold were quantitatively reported.

### Data collection

Parents/caregivers were interviewed to obtain data regarding parental and household smoking (including maternal smoking during pregnancy), demographic data, prematurity, comorbidities, family history of atopy, personal history of atopy, breastfeeding, childcare attendance, existence of older siblings, and total number of household members.

Additionally, for all patients with bronchiolitis, clinical parameters were recorded at admission, including heart rate (HR), respiratory rate (RR), oxygen saturation (SpO_2_), fraction of inspired oxygen (FiO_2_), and Bronchiolitis Score of *Sant Joan de Déu Hospital* (BROSJOD) [18]. Etiological agents were identified from nasopharyngeal swab samples using molecular diagnostic assays (Luminex NxTAG Respiratory Pathogen Panel and FilmArray Respiratory Panel, Discovery Life Sciences, Huntsville, AL, USA). During hospitalization, viral etiology and clinical course data, including the type of respiratory support, need for admission to the pediatric intensive care unit (PICU), and length of hospital stay (LoS), were recorded.

### Measured outcomes

The association between ETS exposure and acute bronchiolitis was assessed by comparing cases and controls. ETS exposure was defined as having ≥ 1 parent or family member in the household who smokes, as reported in interviews, or a serum cotinine level ≥ 2 ng/mL (positive cotinine). In the absence of reference thresholds for high exposure, the degree of exposure was categorized using the median serum cotinine concentration among participants with detectable cotinine levels: high exposure (> median), moderate exposure (≤ median), and low or undetectable exposure (< 2 ng/mL).

During prospective follow-up of participants with bronchiolitis, the association between serum cotinine concentration and disease severity was evaluated according to clinical parameters, PICU admission, and prolonged LoS.

### Statistical analysis

Data were analyzed using SPSS version 29.0.1.0 (IBM Corp., Armonk, NY, USA). Continuous variables were compared using Student’s *t*-test or analysis of variance if they were normally distributed; otherwise, the Mann–Whitney U or Kruskal–Wallis tests were used. Categorical comparisons were performed using Fisher’s exact test or the chi-squared test.

Bivariate and multivariate logistic regression analyses were performed to examine the following: the association between ETS exposure and acute bronchiolitis among hospitalized children (cases vs. controls); and the possible relationship between ETS exposure and both PICU admission and prolonged LoS within the bronchiolitis group. Prolonged LoS was defined as a duration exceeding the 75th percentile of the hospital stays distribution of the cohort. A backward stepwise selection method based on the likelihood ratio was used, and the results are presented as OR with corresponding 95% confidence intervals (CI). Variable selection for inclusion in the multivariate models was based on clinical and statistical criteria (p < 0.2, bivariate analysis). The best models were selected using the Akaike Information Criterion (AIC), and their discriminative ability and predictive performance were evaluated using receiver operating characteristic (ROC) curves with the estimated area under the ROC curve (AUC). Internal validation was performed using stratified five-fold cross-validation, with the model-development procedure repeated within each training fold and discrimination assessed using out-of-fold predicted probabilities. Potential collinearity was assessed using the variance inflation factor (VIF); if collinearity was detected, the most clinically relevant variable was retained in the multivariate model. Differences with p < 0.05 were considered to be statistically significant.

## Results

Among a total of 186 eligible bronchiolitis cases and 123 eligible controls, 151 and 100, respectively, were ultimately enrolled. Non-enrollment was due to parental refusal (9 cases and 6 controls), inability to approach families for recruitment during periods of high clinical workload (23 cases and 11 controls), or an insufficient blood sample for cotinine analysis (3 cases and 6 controls) (Figure 1). Characteristics of the study population are summarized in Table 1. The median age of the cohort was 2.7 months (interquartile range [IQR]: 1.2–7 months) and the median weight at admission was 5.3 kg (IQR: 4–7.3 kg). Significant differences between cases and controls were observed in terms of age, presence of older siblings, number of household members, and family history of atopy, all of which are known factors associated with acute bronchiolitis.

**Figure 1:**
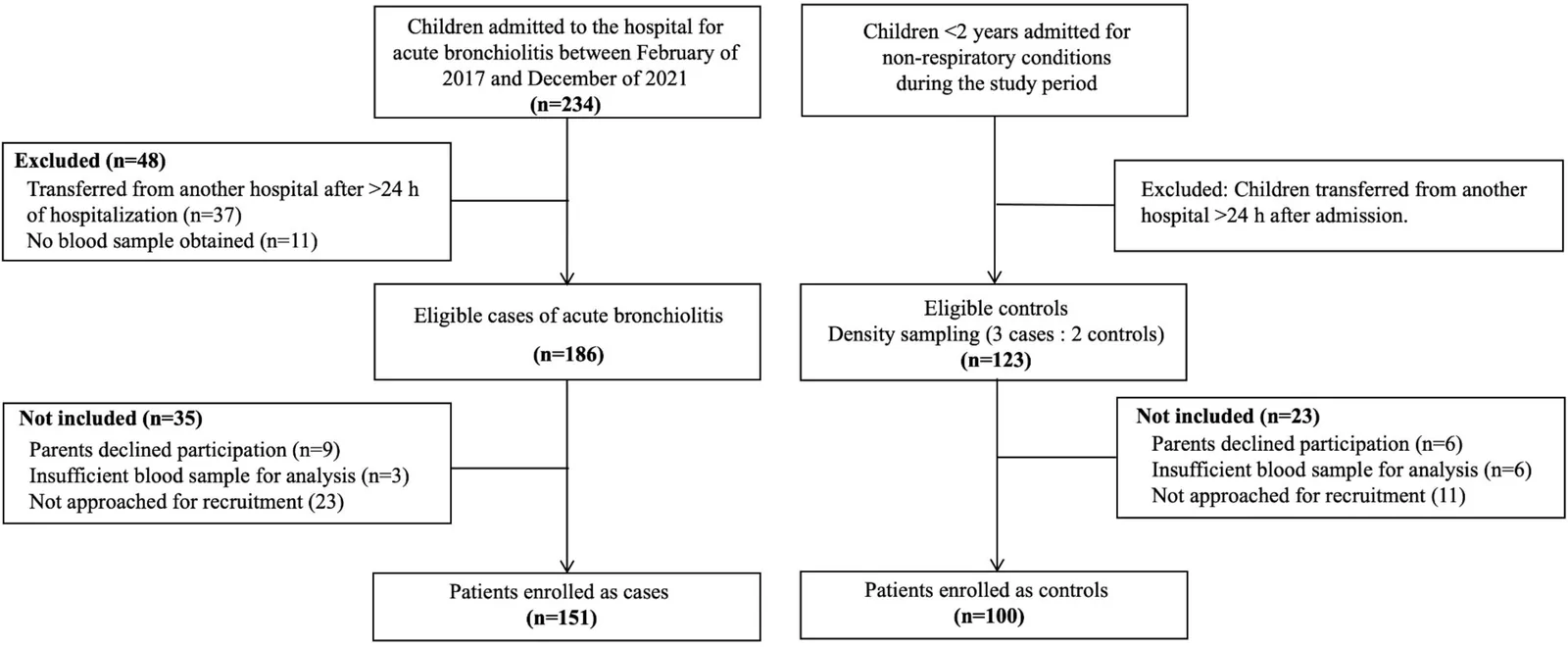
Flow-diagram illustrating the patient selection process.

**Table 1:** Baseline characteristics of cases and controls.

	Total n = 251 (%)	Bronchiolitis n = 151 (%)	Control group n = 100 (%)	*p-value*
Maternal age (years)	34 [29–37]	33 [28–7]	34 [29–37]	*0.357*
a Ethnicity				*0.885*
European (Caucasian)	229 (91.2)	137 (90.7)	92 (92)	
Latin American	9 (3.6)	5 (3.3)	4 (4)	
North Africans (Morocco)	11 (4.4)	8 (5.3)	3 (3)	
South Asians (India/Pakistan)	2 (0.8)	1 (0.7)	1 (1)	
Sex (male)	135 (53.8)	80 (53)	55 (55)	*0.753*
Gestational age (weeks)	39 [38–40]	39 [37–40]	39 [38–40]	*0.718*
Birth weight (Kg)	3.12 [2.75–3.47]	3.18 [2.75–3.53]	3.10 (2.76–3.43)	*0.170*
Weight (kg)	5.25 [4–7.33]	5.17 [4–6.87]	5.79 [4.03–8.16]	*0.237*
Age (months)	2.73 [1.22–7.02]	2.46 [1.22–4.81]	4.23 [1.22–9.11]	*0.039*
Mode of delivery (caesarean section)	81 (32.3)	54 (35.8)	27 (27)	*0.146*
b Underlying disease	17 (6.8)	7 (4.6)	10 (10)	*0.098*
Cardiopathy	4 (1.6)	3 (2)	1 (1)	*0.541*
Bronchopulmonary dysplasia	4 (1.6)	1 (0.7)	3 (3)	*0.148*
Neurological disorders	7 (2.8)	3 (2)	4 (4)	*0.343*
Immunodeficiency	1 (0.4)	1 (0.7)	0	*1*
Others	2 (0.8)	0	2 (2)	*0.158*
Preterm birth (< 37 weeks)	43 (17.1)	24 (15.9)	19 (19)	*0.499*
Siblings	157 (62.5)	109 (72.2)	48 (48)	*<0.001*
c Preexisting allergies	34 (13.5)	22 (14.6)	12 (12)	*0.560*
Atopic dermatitis	18 (7.2)	13 (8.6)	5 (5)	*0.278*
d Family history of atopy	88 (35.1)	67 (44.4)	21 (21)	*<0.001*
Childcare center	26 (10.4)	13 (8.6)	13 (13)	*0.240*
Breastfeeding	138 (55)	86 (57)	52 (52)	*0.491*
Number of household members	4 [3–4]	4 [3–5]	3 [3–4]	*<0.001*

Categorical variables are expressed as absolute value and percentage (%). Quantitative variables are expressed as median and interquartile range [IQR]. No data missing.

a Ethnicity was assigned according to maternal country of origin.

b Some patients present with more than one condition.

c Preexisting allergies: atopic dermatitis, food allergy, or drug allergy.

d Family history of atopy: asthma, allergic rhinitis, food allergy, or drug allergy in parents or siblings.

Diagnoses among children in the control group were distributed as follows: acute pyelonephritis (n = 29); cellulitis or other soft-tissue infections (n = 14); seizures (n = 10); acute gastroenteritis and dehydration (n = 10); sepsis (n = 8); meningitis (n = 5); urgent abdominal surgical conditions (n = 5); febrile syndrome without a focus (n = 3); anemia (n = 3); urgent neurosurgical conditions (n = 2); jaundice (n = 2); and others (n = 9).

### Tobacco use data from parental interviews

Parental interviews revealed that 113 (45%) children were exposed to ETS through parental smoking or other household members. The prevalence of ETS exposure was significantly higher among the bronchiolitis group than that in controls (54.3%vs. 31.0%; p < 0.001), and maternal smoking during pregnancy was reported in 19.2% of cases vs. 6.0% of controls (p = 0.003) (Table 2).

**Table 2:** Comparison of household and parental smoking in cases and controls.

	Total n = 251 (%)	Bronchiolitis n = 151 (%)	Control group n = 100 (%)	*p-value*
Mother smoked during pregnancy	35 (13.9)	29 (19.2)	6 (6)	*0.003*
Maternal cigarettes/day during pregnancy	4 [3 – 5]	4 [3 – 5]	3.5 [2 – 4]	*0.439*
Mother currently smokes	48 (19.1)	34 (22.5)	14 (14)	*0.093*
Current maternal cigarette/day	5 [4–7.5]	5 [3 – 10]	5 [4 – 6]	*0.590*
Father currently smokes	88 (35.1)	66 (43.7)	22 (22)	*<0.001*
Current paternal cigarette/day	10 [5–20]	10 [5–20]	10 [5–20]	*0.845*
Only one of the parent smokes	106 (42.2)	76 (50.3)	30 (30)	*0.001*
Both parents are smokers	30 (12)	24 (15.9)	6 (6)	*0.018*
Another household member smokes	20 (8)	18 (11.9)	2 (2)	*0.004*
Parental and household ETS exposure	113 (45)	82 (54.3)	31 (31)	*<0.001*
Positive cotinine (≥ 2 ng/mL); n (%) Median [IQR]	79 (31.5) 5 [3–14]	55 (36.4) 5 [3–19]	24 (24) 3,5 [3–7]	*0.038* *0.207*
ETS exposure by cotinine level				*0.043*
Low/undetectable exposure (< 2 ng/mL)	172 (68.5)	96 (63.6)	76 (76)	
Moderate exposure (2–5 ng/mL)	46 (18.3)	29 (19.2)	17 (17)	
High exposure (> 5 ng/mL)	33 (13.1)	26 (17.2)	7 (7)	
ETS exposure (questionnaire and/or cotinine +)	132 (52.6)	90 (59.6)	42 (42)	*0.007*

Categorical variables are expressed as absolute value and percentage (%). Quantitative variables are expressed as median and interquartile range [IQR]. No data missing.

### Serum cotinine measurement as a biomarker of passive smoking

Serum cotinine concentrations were measured during the first hours of hospitalization. The median interval between patient arrival to the emergency department and blood sample collection was 3 h (IQR 2–8 h). Cotinine was detected in 79 of 251 children (31.5%), with a median level of 5 ng/ml (IQR 3–14 ng/ml). Serum cotinine was positive in 53.1% of children who reported household smoking and 13.8% of children whose parents denied smoking.

A higher percentage of patients with bronchiolitis exhibited positive cotinine results than controls (36.4%vs. 24%; p = 0.038). When ETS exposure was categorized according to median serum cotinine concentration, high exposure (cotinine > 5 ng/mL) was significantly more prevalent among children with bronchiolitis (17%vs 7%) (Table 2).

### Relationship between ETS exposure and acute bronchiolitis

Based on data obtained from interviews and serum cotinine measurements, 132 (52.6%) children were confirmed to have been exposed to ETS. The prevalence of ETS exposure was higher among the bronchiolitis group than that in the control group (59.6%vs. 42.0%; OR: 2.04; 95% CI: 1.22–3.44; p = 0.007).

In the final multivariable logistic regression model, ETS exposure (OR: 1.93; 95% CI: 1.04–3.56) and prenatal maternal smoking (OR: 3.49; 95% CI: 1.22–9.95) were independently associated with hospitalization for acute bronchiolitis compared with hospitalization for non-respiratory conditions. Additional independent predictors included age (OR 0.92; 95% CI 0.87–0.97), birth weight (OR 1.95; 95% CI 1.19–3.19), cesarean delivery (OR 2.58; 95% CI 1.31–5.06), family history of atopy (OR 3.34; 95% CI 1.77–6.31), and the presence of siblings (OR 2.60; 95% CI 1.44–4.70) (Supplementary Material 1).

### Characteristics and clinical course of patients with acute bronchiolitis

Regarding the etiology of bronchiolitis, RSV was detected alone or in combination with other viruses in 81.5% of cases, and viral co-infections were identified in 47% of cases. The median BROSJOD score at admission was 7 points (IQR 6–9 points). Forty-five (29.8%) infants required admission to the PICU, of whom 39 (86.7%) received noninvasive ventilation, and 4 (2.6%) received invasive mechanical ventilation (IMV). The median LoS was 6 days (IQR 4–10), and 33 children (21.9%) had prolonged LoS (> 10 days).

### Association between cotinine concentration and bronchiolitis progression

When ETS exposure was categorized according to serum cotinine concentration among the 151 infants with bronchiolitis, 26 (17.2%) had high exposure (> 5 ng/mL), 29 (19.2%) had moderate exposure (2–5 ng/mL), and 96 (63,6%) were classified as having low/undetectable exposure (< 2 ng/mL).

Children with high exposure experienced a more severe clinical course. On admission, they exhibited a higher HR, required greater FiO₂, experienced more frequent apneas, and demonstrated increased rates of bacterial infection and antibiotic use. Overall, this high-exposure group required IMV more often and experienced longer LoS (Table 3).

**Table 3:** Demographic and clinical characteristics of patients with acute bronchiolitis by serum cotinine level.

	Low/undetectable exposure (< 2 ng/mL) n = 96	Moderate exposure (2–5 ng/mL) n = 29	High exposure (> 5 ng/mL) n = 26	*p-value*
Maternal age (years)	35 [30–38]	29 [20–33]	32.5 [28.8–36.3]	*<0.001*
Ethnicity				*0.234*
European (Caucasian) Other	85 (88.5) 11 (11.5)	26 (89.7) 3 (10.3)	26 (100) 0	
Sex (male)	50 (52.1)	17 (58.6)	13 (50)	*0.781*
Birth weight (Kg)	3.29 [2.89–3.56]	3.23 [2.69–3.67]	2.95 [2.65–3.12]	*0.041*
Age at admission (months)	2.53 [1.18–5.29]	2.66 [1.38–4.33]	1.75 [0.99–4.32]	*0.520*
Weight at admission (kg)	5.1 [4–7]	5.3 [4.3–7]	4.5 [3.8–5.7]	*0.262*
Underlying disease	4 (4.2)	2 (6.9)	1 (3.8)	*0.811*
Number of household members	4 [3–4]	4 [4–5]	4 [4–5]	*0.078*
Fever (> 38 °C)	58 (60.4)	20 (68.9)	18 (69.2)	*0.613*
Apnea	8 (8.3)	0	7 (26.9)	*0.003*
Food refusal	59 (61.5)	19 (65.5)	18 (69.2)	*0.858*
a Physiological parameters				
SpO2; median (IQR)	96 [93–97]	95 [92–97]	96.5 [94–97]	*0.236*
FiO2; median (IQR)	21 [21–24]	21 [21–22]	24 [21–31.5]	*0.002*
RR (breaths/min); mean (SD)	53.4 (11.4)	55 (8.1)	50 (10.6)	*0.236*
HR (beats/min); mean (SD)	154.8 (18.8)	149.5 (22.7)	164.3 (19.9)	*0.010*
b BROSJOD Score	7 [6–9]	7 [6–8]	8 [6–10]	*0.706*
Maximum FiO2	28 [24–35]	24 [23–29]	30 [24–40]	*0.046*
Maximum C reactive Protein (mg/dL)	0.96 [0.23–5.18]	0.9 [0.26–3.25]	1.78 [0.33–5.4]	*0.458*
Admission blood count				
Leukocytes (10^3^/μL); mean (SD)	11.37 (4.39)	12.49 (3.57)	12.48 (5.51)	*0.398*
Neutrophils (10^3^/μL); mean (SD)	5.05 (3.51)	4.68 (2.55)	5.54 (3.81)	*0.616*
Lymphocytes (10^3^/μL); mean (SD)	5.16 (2.07)	6.62 (2.64)	5.75 (2.62)	*0.026*
Eosinophils(10^3^/μL); median (IQR)	0.06 [0.01–0.9]	0.05 [0.02–0.09]	0.02 [0.01–0.172]	*0.373*
Viral coinfections	46 (47.9)	15 (51.7)	10 (38.5)	0.591
c Bacterial infection	8 (8.3)	2 (6.9)	7 (26.9)	*0.031*
Sepsis	5 (5.2)	1 (3.4)	4 (15.4)	*0.148*
Antibiotic treatment	23 (24)	5 (17.2)	16 (61.3)	*<0.001*
Highest respiratory support				*0.027*
None	2 (2.1)	1 (3.4)	2 (7.7)	
LFNC oxygen therapy	32 (33.3)	16 (55.2)	7 (26.9)	
HFNC oxygen therapy	32 (33.3)	9 (31)	7 (26.9)	
NIV	29 (30.2)	3 (10.3)	7 (26.9)	
IMV	1 (1)	0	3 (11.5)	
PICU admission	31 (32.3)	3 (10.3)	11 (42.3)	*0.020*
PICU stay (days) (n = 45)	5 [4–6]	3 [2.5–8.5]	7 [5.5–10]	*0.027*
LOS (days)	6 [4–9]	6 [4–8.5]	9 [5–12.5]	*0.017*

Categorical variables are expressed as absolute value and percentage (%). Quantitative variables are expressed as mean and standard deviation (SD) or median and interquartile range [IQR].

LFNC, Low-flow nasal cannula oxygen therapy; HFNC, High-flow nasal cannula oxygen therapy; NIV, Non- invasive ventilation; IMV, Invasive mechanical ventilation; PICU, Paediatric Intensive Care Unit; LOS, Length of hospital stay (days).

a At admission.

b *BROSJOD Score* Bronchiolitis Score of Sant Joan de Déu Hospital (0–5 mild; 6–10 moderate; 11–16 severe);.

c Bacterial infection: urinary tract infection or sepsis (confirmed culture-positive).

Factors associated with a prolonged LoS (> 10 days) and PICU admission were also assessed (Supplementary Materials 2 and 3). Multivariate analyses were performed, and optimal predictive models were selected (Figure 2).

**Figure 2:**
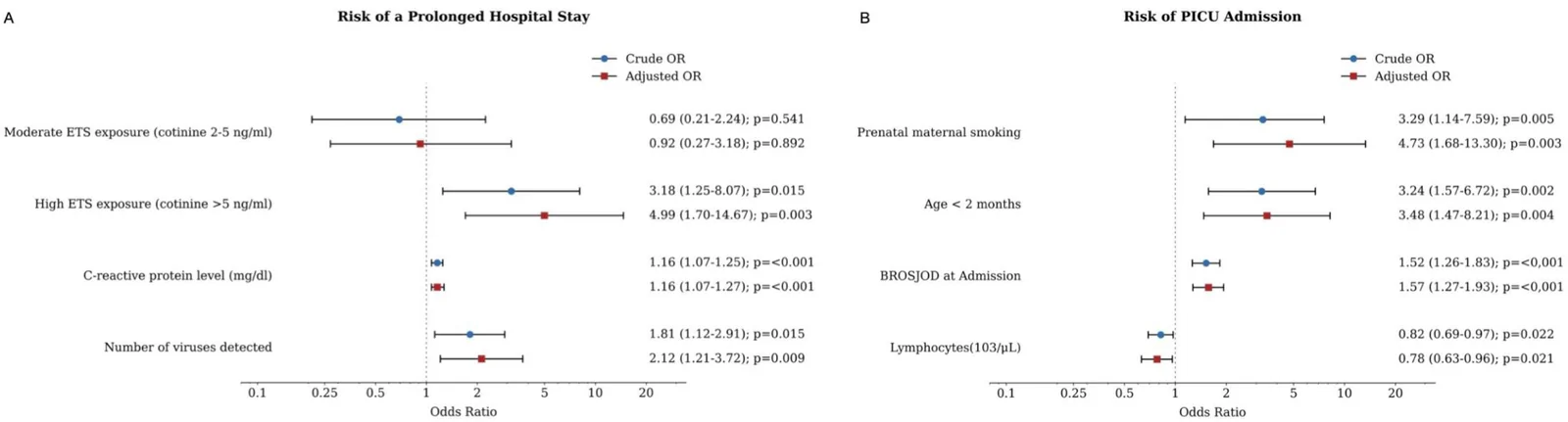
**Multivariable logistic regression models for prolonged length of stay (LoS) and pediatric intensive care unit (PICU) admission.** Crude and adjusted odds ratios (ORs) and 95% confidence intervals (CIs) are shown. (A) Prolonged LoS (> 10 days): AIC = 138.9; model AUC = 0.799 (95% CI: 0.720–0.879); five-fold cross-validated AUC = 0.756 (95% CI: 0.662–0.849). (B) PICU admission: AIC = 148.1; model AUC = 0.840 (95% CI: 0.770–0.910); five-fold cross-validated AUC = 0.824 (95% CI: 0.755–0.892).

#### Predictors of prolonged los

High ETS exposure (cotinine > 5 ng/mL) independently predicted a prolonged LoS (OR: 4.99; 95% CI: 1.70–14.67). Other independent predictors included elevated C-reactive protein level (OR: 1.16; 95% CI: 1.07–1.27) and viral coinfection, whereby each additional virus detected doubled the odds of prolonged hospital stay (OR: 2.12; 95% CI: 1.21–3.72) (Figure 2A). In a retrospective analysis based on the observed effect in the model, the statistical power for the association between high ETS exposure and prolonged hospital stay was 83.2%.

#### Predictors of admission to the PICU

ETS exposure and serum cotinine concentration were not associated with an increased risk of PICU admission. However, prenatal maternal smoking emerged as an independent predictor of PICU admission (OR: 4.73; 95% CI: 1.68–13.3). Additional prognostic factors included age under 2 months (OR: 3.48; 95% CI: 1.47–8.21), a higher severity score at admission (BROSJOD) (OR: 1.57; 95% CI: 1.27–1.93), and lower lymphocyte levels (OR: 0.78; 95% CI: 0.63–0.96) (Figure 2B). The statistical power estimated from the observed effect for the association between prenatal maternal smoking and PICU admission was 83.6%.

## Discussion

In the present study, ETS exposure was more prevalent among children hospitalized with acute bronchiolitis than among controls admitted for non-respiratory conditions. Among children with bronchiolitis, higher ETS exposure was also associated with a worse clinical course. Moreover, serum cotinine levels > 5 ng/mL were correlated with more severe symptoms and markedly increased the risk for prolonged LoS. Furthermore, prenatal maternal smoking was associated with acute bronchiolitis and was identified as an independent predictor of PICU admission.

In this study, more than one-half of the children were exposed to ETS. The combined use of parental interviews and serum cotinine measurements enabled a more accurate estimation of ETS exposure in children. Parental interviews indicated that 45% of the children were exposed to domestic smoking. However, when serum cotinine screening data were incorporated, the prevalence of exposure increased to 53%, which was significantly higher in the bronchiolitis group than that among controls. After adjustment for potential confounders, ETS exposure remained associated with bronchiolitis case status (OR: 1.93; 95% CI: 1.04–3.56). This finding is consistent with the study by Cano Fernández et al., [17] who found that household smoking is significantly associated with the development of bronchiolitis (OR: 2.2; 95% CI: 1.39–3.45), showing an effect size similar to that obtained in the present study. Exposure to ETS has typically been investigated through parental interviews. Although widely used, this approach may underestimate actual exposure due to social stigma and fear of negative judgment among smoking parents [19]. Consistent with this, the present study identified children with detectable serum cotinine levels despite parental reports of no household smoking.

The objective assessment of ETS exposure is challenging. Although blood cotinine is widely recognized as a reliable biomarker of tobacco smoke exposure, [13,20,21] urine and saliva are also well-established non-invasive alternatives [22]. In the present study, serum was selected because blood samples were already being obtained for clinical purposes, allowing cotinine measurement without additional venipuncture; moreover, salivary cotinine analysis was not available at the laboratory where the samples were analyzed. Urinary cotinine was considered less suitable for this population because spot concentrations are affected by urine dilution and may require normalization to urinary creatinine or specific gravity, while sample collection can be technically challenging in non-toilet-trained infants [22]. Despite its practical advantages and its established value as a biomarker of tobacco smoke exposure, serum cotinine remains subject to several limitations. Cotinine metabolism varies with age, genetic polymorphisms, and other individual characteristics, and its 16–20 h half-life limits the ability to capture exposure over longer time periods [22]. In the present study, blood samples were obtained several hours after emergency department presentation within a smoke-free hospital environment, which may have reduced cotinine levels and hindered the identification of low-intensity, intermittent, or more remote exposures. This potential underestimation may also have been accentuated by the relatively high laboratory-established detection limit (2 ng/mL). No single modality can capture the full spectrum of exposure to ETS. Therefore, as a more robust approach, the authors recommend combining questionnaire data with biomarker measurements to achieve a more comprehensive and accurate exposure evaluation.

Although no universally accepted serum cotinine cut-off has been established for defining high-intensity passive ETS exposure in children, values ranging approximately from 3 to 10 ng/mL have been reported in previous pediatric studies [6,23,24]. The > 5 ng/mL threshold used in the present study was not intended to represent a validated clinical cut-off; rather, it corresponded to the median serum cotinine concentration among participants with detectable levels. Although necessarily arbitrary in the absence of a validated reference value, this data-driven categorization was consistent with previous research and allowed us to move beyond a simple exposed/non-exposed classification to explore whether higher levels of exposure were associated with clinically relevant differences in disease severity. In the present study, infants with bronchiolitis and serum cotinine concentrations > 5 ng/mL had a higher HR, more frequent apnea and bacterial coinfections, increased FiO_2_ requirements, a greater need for IMV, and prolonged LoS. In this regard, Maedel et al. [6] found that children with respiratory infections associated with RSV and urinary cotinine ≥ 7 ng/mL exhibited lower SpO_2_ and higher severity scores at admission; however, LoS, and the risks for IMV and PICU admission were not evaluated. In this context, the present study showed that a serum cotinine level > 5 ng/mL independently predicted a prolonged LoS, with nearly fivefold higher odds of hospitalization exceeding 10 days. To our knowledge, this is the first study to demonstrate that objectively measured intense ETS influences LoS among patients with bronchiolitis. However, the absence of a consistent monotonic exposure-response pattern across all outcomes, possibly related to the small and unequal sizes of the exposure groups, limits inference of a dose-response relationship.

Furthermore, these findings reinforce the premise that tobacco-related harm begins in utero [9,17,25]. Interview data indicated that 13.5% of the mothers smoked during pregnancy; prenatal maternal smoking was not only significantly associated with hospitalization for bronchiolitis but also emerged as an independent predictor of severity, increasing the risk of PICU admission 4.7-fold. These results are consistent with the findings of a multicenter study by Stevenson et al., [7] who identified an independent relationship between prenatal exposure to ETS and the need for intensive care.

The different clinical outcomes associated with prenatal and postnatal tobacco exposure may partly reflect differences in the timing and biological effects of exposure. Prenatal maternal smoking was associated with PICU admission, whereas current high-intensity ETS exposure (serum cotinine > 5 ng/mL), was associated with prolonged LoS. Prenatal exposure occurs during a critical period of lung development and may impair pulmonary growth and immune responses, potentially increasing vulnerability to severe respiratory compromise [7,26]. In contrast, recent postnatal ETS exposure may promote airway inflammation and impair mucociliary clearance, [27] which could contribute to a more prolonged clinical course. These mechanisms may provide a plausible biological explanation for these findings, although the design of this study does not allow causal inference.

The present study has several limitations. First, its single-center design may limit the generalizability of the findings. Because the results reflect local sociodemographic profiles and healthcare practices, they should be interpreted with caution and validated in multicenter cohorts. Second, the use of hospitalized children with non-respiratory conditions as controls may have introduced selection bias, particularly because some infectious conditions may themselves be associated with ETS exposure. Although respiratory diagnoses were excluded, individual control diagnoses were not screened for potential associations with ETS; therefore, some influence on the estimated case-control association cannot be excluded. Third, socioeconomic and educational variables were not collected, which may have introduced unmeasured confounding. This decision sought to minimize the respondent burden and enhance acceptability by prioritizing clinical data collection. In this context, Semple et al., [28] demonstrated that while socioeconomic deprivation correlates strongly with domestic smoking prevalence, this association does not extend to the severity of bronchiolitis once hospitalized. Nevertheless, the authors acknowledge the importance of including these variables in future research. Finally, despite adjustment for relevant baseline differences between cases and controls in the multivariable analysis, residual confounding inherent to the observational case-control design cannot be excluded. Therefore, the observed associations should be interpreted cautiously and should not be considered evidence of causality.

Notwithstanding these limitations, this study has several notable strengths. A key asset was the inclusion of serum cotinine as an objective biomarker, which enabled a more accurate assessment of ETS exposure in children and reduced the risk of misclassification. In addition, the low rate of parental refusal minimized participation bias and enhanced the validity and generalizability of the findings. The prospective follow-up of children with bronchiolitis also enabled a detailed evaluation of multiple clinical outcomes according to ETS exposure level, an aspect rarely examined in previous research.

In conclusion, prenatal maternal smoking and postnatal ETS exposure were associated with acute bronchiolitis and greater disease severity among hospitalized children. Despite existing tobacco control policies, passive exposure to tobacco smoke among children remains an avoidable public health concern. Preventive strategies should be strengthened across all levels of care, including family education on the harmful effects of exposure to tobacco smoke and, when feasible, using objective biomarkers to improve the detection and quantification of risk to children

## Authors’ contributions

(1) The conception and design of the study: Lorena Bermúdez-Barrezueta, Wysali Trapiello Fernández, María de la Asunción Pino-Vázquez, Hermenegildo González García. (2) Acquisition of data: Wysali Trapiello Fernández, Irune Torres Ballester, Pablo López-Casillas, Pilar Uribe-Reina. (3) Analysis and interpretation of data: Sergio Madero-Juez, Lorena Bermúdez-Barrezueta, María de la Asunción Pino-Vázquez. (4) Drafting the article: Lorena Bermúdez-Barrezueta. (5) Revising the manuscript critically for important intellectual content: Sergio Madero-Juez, María de la Asunción Pino-Vázquez, Wysali Trapiello Fernández, Hermenegildo González García. All authors read and approved the final manuscript.

## Funding

This study was funded by the Ernesto Sánchez Villares Foundation and the Department of Pediatrics, Faculty of Medicine, University of Valladolid. The funders had no role in study design, data collection, data analysis, manuscript preparation, or the decision to publish.

## Ethics approval

The study received approval from the Clinical Research Ethics Committee (protocol no 17–559 CINV 16–146) of the center, in accordance with the principles established in the Declaration of Helsinki. Written informed consent was obtained from the parents or legal guardians of all participants before inclusion.

## Data availability

The data that support the findings of this study are available from the corresponding author.

## Conflicts of interest

The authors declare no conflicts of interest.

## Appendix Supplementary materials

Image, application 1
