# Supplementary material for: Exposure to Environmental Tobacco Smoke: a Modifiable Factor of Hospitalization and Severity of Acute Bronchiolitis
Source: J Pediatr (Rio J). 2026 Oct 9;103(1):101629. doi: 10.1016/j.jped.2026.101629 (PMC13677299; doi:10.1016/j.jped.2026.101629)
Supplement: Supplementary file 1 [file mmc1.docx]

**JPED-D-26-00250_Supplementary Material**

**Supplementary Material 1** Association between ETS exposure and acute bronchiolitis: univariable and multivariable logistic regression analyses.

|  | **Univariate analysis** | | | **Multivariate analysis** | | |
| --- | --- | --- | --- | --- | --- | --- |
|  | **OR** | **95% CI** | ***p-value*** | **OR** | **95% CI** | ***p-*value** |
| Maternal age (years) | 0.98 | 0.94-1.02 | *0.316* |  |  |  |
| Ethnicity (Non-Caucasian) | 1.18 | 0.47-21 | *0.727* |  |  |  |
| Sex (male) | 1.09 | 0.65-1.80 | *0.753* |  |  |  |
| Birth weight (Kg) | 1.38 | 0.93-2.04 | *0.111* | 1.95 | 1.19-3.19 | *0.008* |
| Age at admission (months) | 0.93 | 0.88-0.98 | *0.004* | 0.92 | 0.87-0.97 | *0.005* |
| Weight at admission (kg) | 0.91 | 0.82-1.01 | *0.068* |  |  |  |
| Mode of delivery (caesarean section) | 1.51 | 0.87-2.62 | *0.147* | 2.58 | 1.31-5.06 | *0.006* |
| Underlying disease | 0.44 | 0.16-1.19 | *0.106* |  |  |  |
| Siblings | 2.81 | 1.66-4.78 | *<0.001* | 2.60 | 1.44-4.70 | *0.001* |
| Atopic dermatitis | 1.79 | 0.62-5.19 | *0.284* |  |  |  |
| Family history of atopy | 3.00 | 1.68-5.35 | *<0.001* | 3.34 | 1.77-6.31 | *<0.001* |
| Childcare center | 0.62 | 0.27-1.39 | *0.244* |  |  |  |
| Breastfeeding | 1.22 | 0.74-2.03 | *0.440* |  |  |  |
| Number of household members | 1.59 | 1.17-2.16 | *0.003* |  |  |  |
| Maternal smoking during pregnancy | 3.37 | 1.49-9.34 | *0.005* | 3.49 | 1.22-9.95 | *0.020* |
| Current maternal smoking | 1.79 | 0.90-3.53 | *0.096* |  |  |  |
| Paternal smoking | 2.75 | 1.55-4.89 | *<0.001* |  |  |  |
| Both parents smoking | 2.96 | 1.16-7.53 | *0.023* |  |  |  |
| Positive cotinine (≥2 ng/mL) | 1.81 | 1.03-3.20 | *0.039* |  |  |  |
| ETS exposure by cotinine level  Low/undetectable exposure (<2 ng/mL)  Moderate exposure (2-5 ng/mL)  High exposure (>5 ng/mL) | Ref.  1.35  2.94 | 0.69-2.64  1.21-7.14 | *0.051*  *0.379*  *0.017* |  |  |  |
| Overall ETS exposure | 2.04 | 1.22-3.40 | *0.007* | 1.93 | 1.04-3.56 | *0.037* |

**Variables entered into the initial multivariable model:** birth weight, age at admission, mode of delivery, underlying disease, family history of atopy, siblings, number of household members, maternal smoking during pregnancy, and overall ETS exposure. The final model retained all except underlying disease and number of household members.

Weight at admission was excluded because of collinearity with age, and individual current household smoking variables because of collinearity with overall ETS exposure. Positive cotinine (≥2 ng/mL) was not entered separately because it is a component of the composite ETS exposure variable. Maternal smoking during pregnancy was retained as a distinct prenatal exposure and showed no relevant collinearity with overall ETS exposure.

AIC= 287.77. Model AUC: 0.777 (95% CI 0.719–0.835). Internal validation using stratified five-fold cross-validation yielded an AUC of 0.765 (95% CI 0.706–0.825).

**Supplementary Material 2** Characteristics of patients with bronchiolitis by prolonged LOS.

|  | **Prolonged Hospital Stay** | | |
| --- | --- | --- | --- |
|  | **Yes (n=33)** | **Not (n=118)** | ***p-value*** |
| Maternal age (years) | 31 [27-36] | 33.5 [29-38] | *0.120* |
| Sex (male) | 19 (57.6) | 61 (51.7) | *0.550* |
| Birth weight (Kg) | 2.99 (0.642) | 3.15 (0.628) | *0.210* |
| Age at admission (months) | 1.77 [0.99-3.68] | 2.68 [1.27-5.19] | *0.002* |
| Weight at admission (kg) | 4.55 [3.72-5.33] | 5.40 [4.18-7.03] | *<0.001* |
| Mode of delivery (caesarean section) | 13 (39.4) | 41 (34.7) | *0.622* |
| Preterm birth (<37 weeks) | 6 (18.2) | 18 (15.3) | *0.684* |
| Underlying disease | 4 (12.1) | 3 (2.5) | *0.021* |
| Siblings | 24 (72.7) | 85 (72) | *0.937* |
| Preexisting allergies | 6 (18.2) | 16 (13.6) | *0.518* |
| Family history of atopy | 14 (42.4) | 53 (44.9) | *0.799* |
| Childcare center | 2 (6.1) | 11 (9.3) | *0.555* |
| Breastfeeding | 19 (57.6) | 67 (56.8) | *0.935* |
| Number of household members | 4 [3-5] | 4 [3-4] | *0.679* |
| Fever (>38ºC) | 24 (72.7) | 72 (67.9) | *0.237* |
| Apnea | 6 (18.2) | 9 (7.6) | *0.073* |
| ^a^BROSJOD Score | 7 [5-9] | 7 [6-9] | *0.314* |
| ^a^Physiological parameters  SpO2; median [IQR]  FiO2; median [IQR]  RR (breaths/min); median [IQR]  HR (beats/min); mean (SD) | 96 [94–97]  24 [21–27.5]  55 [45-60]  156.7 (17.2) | 95.5 [93–97]  21 [21–23]  54 [46-60]  155.1(20.9) | *0.337*  *0.006*  *0.605*  *0.676* |
| Sepsis | 7 (21.2) | 3 (2.5) | *<0.001* |
| ^b^Bacterial infection | 11 (33.3) | 6 (5.1) | *<0.001* |
| Maximum C reactive Protein (mg/dL) | 6.32 [1.46-9.5] | 0.74 [0.22-2.69] | *<0.001* |
| Admission blood count*; mean (SD)*  Leukocytes (10^3^/μL)  Neutrophils (10^3^/μL)  Lymphocytes (10^3^/μL)  Eosinophils(10^3^/μL) | 11.9 (5.69)  5.69 (4.21)  5.0 (2.22)  0.117 (0.161) | 11.69 (4.08)  4.88 (3.13)  5.65 (2.32)  0.95 (0.116) | *0.546*  *0.382*  *0.022*  *0.782* |
| Etiology  RSV  Rhinovirus/enterovirus  Adenovirus  Metapneumovirus  Parainfluenza  Influenza  Bocavirus  Coronavirus | 28 (84.8)  14 (42.4)  2 (6.1)  2 (6.1)  2 (6.1)  2 (6.1)  4 (12.1)  6 (18.2) | 95 (80.5)  35 (29.7)  10 (8.5)  5 (4.2)  7 (5.9)  4 (3.4)  12 (10.2)  11 (9.3) | *0.571*  *0.166*  *0.650*  *0.660*  *0.978*  *0.487*  *0.747*  *0.155* |
| Viral coinfections | 20 (60,6) | 51 (43,2) | *0,077* |
| Number of viruses detected | 2 [1-3] | 1 [1-2] | *0,030* |
| Maternal smoking in pregnancy | 12 (36.4) | 17 (14.4) | *0.005* |
| Current maternal smoking | 12 (36.4) | 22 (18.6) | *0.031* |
| Paternal smoking | 19 (57.6) | 47 (39.8) | *0.069* |
| Both parents smoking | 11 (33.3) | 13 (11) | *0.002* |
| Positive cotinine (≥ 2 ng/mL) | 15 (45.5) | 40 (33.9) | *0.223* |
| ETS exposure by cotinine level  Low/undetectable exposure (<2 ng/mL)  Moderate exposure (2-5 ng/mL)  High exposure (>5 ng/mL) | 18 (54.5)  4 (12.1)  11 (33.3) | 78 (66.1)  25 (21.2)  15 (12.7) | *0.018* |
| Overall ETS exposure | 22 (66.7) | 68 (57.6) | *0.350* |

Categorical variables are expressed as absolute value and percentage (%). Quantitative variables are expressed as mean and standard deviation (SD) or median and interquartile range [IQR] if they were not normally distributed.

^a^At admission. ^b^Bacterial infection: urinary tract infection or sepsis (confirmed culture-positive).

**Supplementary Material 3** Characteristics of patients with bronchiolitis by PICU admission.

|  | **PICU Admission** | | |
| --- | --- | --- | --- |
|  | **Yes (n=45)** | **Not (n=106)** | ***p-value*** |
| Maternal age (years) | 33 [27.5-36.5] | 33 [28.5-37.5] | *0.845* |
| Sex (male) | 24 (53.3) | 56 (52.8) | *0.995* |
| Birth weight (Kg) | 3.19 (658) | 3.08 (622) | *0.448* |
| Age at admission (months) | 1.6 [0.91- 4.42] | 2.7 [1.3-4.88] | *0.053* |
| Weight at admission (kg) | 4.55 [3.48-7.03] | 5.39 [4.26-7.03] | *0.016* |
| Mode of delivery (caesarean section) | 18 (40) | 36 (34) | *0.479* |
| Preterm birth (<37 weeks) | 5 (11.1) | 19 (17.9) | *0.295* |
| Underlying disease | 4 (8.9) | 3 (2.8) | *0.105* |
| Siblings | 36 (80) | 73 (68.9) | *0.163* |
| Preexisting allergies | 6 (13.3) | 16 (15.2) | *0.763* |
| Family history of atopy | 17 (37.8) | 50 (47.2) | *0.288* |
| Childcare center | 2 (4.4) | 11 (10.4) | *0.235* |
| Breastfeeding | 24 (53.3) | 62 (58.5) | *0.558* |
| Number of household members | 4 [4-5] | 4 [3-5] | *0.568* |
| Fever (>38ºC) | 28 (62.2) | 68 (64.2) | *0,766* |
| Apnea | 11 (24.4) | 4 (3.8) | <0.001 |
| ^a^BROSJOD Score | 9 [7–10] | 6.5 [5–8] | <0.001 |
| ^a^Physiological parameters  SpO2; median [IQR]  FiO2; median [IQR]  RR (breaths/min); median [IQR]  HR (beats/min); mean (SD) | 96 [94–97]  24 [21–30]  60 [48-65]  163.2 (17.8) | 95.5 [92–97]  21 [21–23]  52 [45-60]  152.1(20.3) | *0.366*  *<0.001*  *0.024*  *0.002* |
| Sepsis | 5 (11.1) | 5 (4.7) | 0.148 |
| ^b^Bacterial infection | 10 (22.2) | 7 (6.6) | *0.005* |
| Maximum C reactive Protein (mg/dL) | 2.39 [0.66–8.04] | 0.84 [0.22–3.67] | *0.005* |
| Admission blood count; mean (SD)  Leukocytes (10^3^/μL)  Neutrophils (10^3^/μL)  Lymphocytes (10^3^/μL)  Eosinophils(10^3^/μL) | 11.699 (5.52)  5.6501(4.28)  4.864 (2.22)  0.117 (0.152) | 11.810 (3.96)  4.814 (2.92)  5.833 (2.35)  0.091 (0.114) | *0.904*  *0.546*  *0.020*  *0.680* |
| Etiology  RSV  Rhinovirus/enterovirus  Adenovirus  Metapneumovirus  Parainfluenza  Influenza  Bocavirus  Coronavirus | 40 (88.9)  16 (35.6)  3 (6.7)  1 (2.2)  1 (2.2)  2 (4.4)  4 (8.9)  8 (17.8) | 83 (78.3)  33 (31.1)  9 (8.5)  6 (5.7)  8 (7.5)  4 (3.8)  12 (11.3)  9 (8.5) | *0.126*  *0.595*  *0.705*  *0.353*  *0.206*  *0.847*  *0.657*  *0.099* |
| Viral coinfections | 23 (51,1) | 48 (45,3) | 0,512 |
| Number of viruses detected | 2 (1-2) | 1 (1-2) | 0,317 |
| Maternal smoking in pregnancy | 15 (33.3) | 14 (13.2) | *0.010* |
| Current maternal smoking | 16 (35.6) | 18 (17) | *0.012* |
| Paternal smoking | 17 (37.8) | 49 (46.2) | *0.338* |
| Both parents smoking | 12 (26.7) | 12 (11.3) | *0.018* |
| Positive cotinine (≥ 2 ng/mL) | 14 (31.1) | 41 (38.7) | *0.377* |
| ETS exposure by cotinine level  Low/undetectable exposure (<2 ng/mL)  Moderate exposure (2-5 ng/mL)  High exposure (>5 ng/mL) | 31 (68.9)  3 (6.7)  11 (24.4) | 65 (61.3)  26 (24.5)  15 (14.2) | *0.024* |
| Overall ETS exposure | 23 (51.1) | 67 (63.2) | *0.166* |

Categorical variables are expressed as absolute value and percentage (%). Quantitative variables are expressed as mean and standard deviation (SD) or median and interquartile range [IQR] if they were not normally distributed.

^a^At admission. ^b^Bacterial infection: urinary tract infection or sepsis (confirmed culture-positive).
